# **Effect of Three-Layer Polylactic Acid/Gelatine/Polybutylene Adipate-co-Terephthalate Film with Added *Physalis* Leaf Extract on Shelf-Life Extension of Fish Meat Powder** ^§^

**DOI:** 10.17113/ftb.63.01.25.8553

**Published:** 2025-03

**Authors:** Gokulprasanth Murugan, Soottawat Benjakul, Balasundari Subbiah, Manikandavelu Dhanushkodi, Ganesan Pandi, Elavarsan Govindhasami, Nimish Mol Stephen, Muralidharan Nagarajan

**Affiliations:** 1 Department of Fish Processing Technology, Tamil Nadu Dr. J Jayalalithaa Fisheries University, Dr. M.G.R Fisheries College and Research Institute, Ponneri – 601 204, Tamil Nadu, India; 2 International Center of Excellence in Seafood Science and Innovation (ICE-SSI), Faculty of Agro-Industry, Prince of Songkla University, Hat Yai, Songkhla 90110, Thailand; 3 Tamil Nadu Dr. J Jayalalithaa Fisheries University, Dr. M.G.R Fisheries College and Research Institute, Thalainayeru – 614 712, Tamil Nadu, India; 4 Department of Aquatic Environment Management, Tamil Nadu Dr. J Jayalalithaa Fisheries University, Dr. M.G.R Fisheries College and Research Institute, Ponneri – 601 204, Tamil Nadu, India; 5 Department of Fish Processing Technology, Tamil Nadu Dr. J Jayalalithaa Fisheries University, Fisheries College and Research Institute, Thoothukudi – 628 008, Tamil Nadu, India

**Keywords:** three-layer film, fish skin gelatine, *Physalis* leaf extract, bioplastics, lipid oxidation, volatile compounds

## Abstract

**Research background:**

Nowadays, there is a growing interest in active packaging with the addition of natural extracts due to safety concerns and consumer preferences. *Physalis angulata* is a medicinal and edible species of the Solanaceae family and is rich in phenolic compounds. Phenolic compounds, the secondary metabolites, are synthesised and stored in all plant tissues. They can be used as plasticizers or fillers to improve the interfacial interaction between the two biopolymers and prevent the transfer of moisture and gas from the food product and extend the shelf life to some degree.

**Experimental approach:**

The effect of a three-layer (P/G/B) film based on polylactic acid (P), gelatine (G) and polybutylene adipate-co-terephthalate (B) incorporated or not with *Physalis* leaf extract (PLE) on the quality changes of fish meat powder (sample) stored at 27–30 °C (30 days) was investigated in comparison with control (uncovered), polyethylene (PE), polylactic acid, polybutylene adipate-co-terephthalate and gelatine films. The samples were sealed in a cylindrical bottle covered with the prepared films. The storage properties such as moisture content, pH, peroxide value (PV), thiobarbituric acid reactive substances (TBARS), total volatile basic nitrogen (TVB-N), changes in colour, sensory properties and volatile compounds of samples packed with the developed films were analysed.

**Results and conclusions:**

The moisture content was lower in the sample covered with the P/G/B-PLE-7 % film throughout the storage period. However, the sample covered with PE film had the highest PV (p<0.05). TBARS, TVB-N and volatile compounds decreased in the sample covered with P/G/B-PLE-7 % on day 30 of storage. The incorporation of *Physalis* leaf extract into the three-layer film improved the properties of the film and extended the shelf life of fish meat powder. Thus, the addition of *Physalis* leaf extract to the three-layer film could serve as a biodegradable active packaging and be a promising substitute for commercial plastic films.

**Novelty and scientific contribution:**

This is the first report on the study of chemical changes of fish meat powder covered with a three-layer (P/G/B) film incorporated with *Physalis* leaf extract. This will lead to a better understanding of the role of the three-layer (P/G/B) film containing *Physalis* leaf extract on the extension of shelf life of fish meat powder.

## INTRODUCTION

Food is externally preserved by packaging throughout the supply chain, including storage, transport and distribution. Food, especially meat and meat products, need to be packed and preserved from both intrinsic and extrinsic factors. Food packaging must withstand environmental contamination as well as other factors such as temperature, odour, physical damage, dust, shocks, humidity and microorganisms. Packaging must extend the shelf life of preserved food products and reduce food loss and waste (*1*). The use of traditional chemical preservatives in food production has been reduced due to safety concerns. Nowadays, novel natural additives have attracted great interest as a promising replacement for chemical additives (*2*), especially in the form of active additives incorporated into the packaging.

Gelatine is a highly effective biopolymer known for its exceptional film-forming properties and versatility among animal proteins (*3*, *4*). It can be used as an outstanding material for food packaging as it has high oxygen barrier and UV blocking properties (*5*). Hence, the gelatine packaging films can prevent lipid oxidation in seafoods and further extend the shelf life due to the remarkable oxygen barrier property (*6*). However, the gelatine films have inadequate (mechanical and water vapour permeability (WVP)) barrier properties, mostly due to its hydrophilicity. A recent study has found that the inclusion of copolymers reduced the stiffness and enhanced the barrier properties of gelatine film (*7*).

Polylactic acid is an easily degradable polyester with outstanding attributes such as hydrophobicity, high mechanical strength, biocompatibility and thermal plasticity, comparable to many petroleum-based polymers (*8*). Polylactic acid, a renewable plastic (*9*), has been used in a wide range of fields including commercial packaging, pharmaceutical and biological applications. However, it has certain constraints, most notably weak durability and high brittleness (*10*).

Polybutylene adipate-co-terephthalate is an emerging biodegradable polymer that shows great potential as a substitute for packaging applications. This is attributed to its outstanding processability, exceptional elongation at break, biocompatibility, excellent thermal properties and flexibility (*11*). As a flexible synthetic aliphatic-aromatic copolyester, it has tensile properties comparable to low-density polyethylene (LDPE) while offering excellent thermal and mechanical properties (*12*). However, its commercial use is limited due to its high cost and relatively better water vapour barrier property than conventional films (*13*).

The natural compounds contained in plant extracts and essential oils are important for human health and food preservation (*14*). Natural extracts are thus of great interest to the food industry as potential substitutes for synthetic additives (*15*). The numerous fruit and plant parts of agricultural crops have proven to be a reliable source of fillers in packaging applications. Plant extracts have attracted great interest, as they contain more phenolic compounds with excellent cross-linking, antioxidant and antibacterial functions. Food packaging films developed with the addition of natural extracts had altered barrier properties and bioactivities of the films (*16*). The main purpose of packaging systems is to prevent the transfer of moisture and gases either from or to the product.

An important barrier that measures the resistance of a film to water vapour is water vapour permeability (WVP) (*17*). Reduced water vapour transfer between food (internal) and the environment (external) due to a lower WVP of the packaging is preferable to extend the shelf life of the product (*18*). The incorporation of the extract, which has hydrophobic properties, could increase the overall hydrophobicity of the film, thereby reduce its WVP (*16*). The addition of a polyphenol-rich extract with gelatine nanocomposite films reduced the WVP and moisture content of stored fish meat powder (*19*). Green tea extract in combination with polylactic acid significantly reduced the TVB-N value in salmon (smoked) (*20*). The significant reduction in pH, TBARS and TVB-N was found in golden pompano fillets coated with gelatine film (*21*). The presence of basil leaf essential oil in a gelatine nanocomposite film resulted in a significant reduction in pH, PV, TBARS and TVB-N in sea bass slices (*22*). Thus, the aim of this study focuses on the chemical changes of fish meat powder covered with a three-layer (P/G/B) film with and without *Physalis* leaf extract during a storage period of 30 days (27–30 °C).

## MATERIALS AND METHODS

### Chemicals

Fish skin gelatine, polybutylene adipate-co-terephthalate and polylactic acid were purchased from Vihn Hoan (Dong Thap Province, Vietnam), Jinhui Zhalong High Technology Co., Ltd. (Shanxi, PR China) and NaturTec (Chennai, India), respectively. Trichloroacetic acid and 2*-*thiobarbituric acid were procured from HiMedia Laboratories Pvt. Ltd. (Mumbai, India). Glycerol, chloroform, sodium hydroxide, potassium iodide, sodium thiosulfate, starch, acetic acid, boric acid, perchloric acid, sodium sulfate and hydrochloric acid were obtained from Chemspure Pvt. Ltd. (Chennai, India).

### Ethanolic extraction from Physalis angulata

*Physalis angulata* leaves were collected from a plantation in Ponneri (Tamil Nadu, India), dried at 45 °C for 8 h, milled, sieved and stored in air-tight pouches at 4 °C (*7*, *23*). Leaf powder (10 g) was extracted with 60 % ethanol (1:10, *m*/*V*) by magnetic stirring (Neuation Technologies, Gandhinagar, India) for 3 h (*24*), followed by centrifugation (centrifuge model R-24; Remi, Maharashtra, India) at 3000×*g* for 30 min. The supernatant was filtered, evaporated (Lark innovative Fine Teknowledge, Chennai, India) at 40 °C, purged with nitrogen, lyophilized (SLP Reftech, Chennai, India) and powdered to obtain *Physalis* leaf extract (PLE).

### Preparation of three-layer film with incorporated Physalis leaf extract

Three-layer (P/G/B) films were prepared using fish gelatine and bioplastics (polylactic acid and polybutylene adipate-co-terephthalate) at 4 % (*m*/*V*) (*m*(total solid)=4 g containing 0.8 g polylactic acid, 2.4 g fish gelatine and 0.8 g polybutilene adipate-co-terephthalate) as defined by Murugan *et al.* (*7*). Film samples with and without incorporation of 7 % of *Physalis* leaf extract in each layer were named P/G/B-PLE-7 % and P/G/B-PLE-0 %, respectively. The films with polylactic acid on the exposed side was referred to as P/G/B-PLE-0 % and P/G/B-PLE-7 % and the films with polybutylene adipate-co-terephthalate on the exposed side were named B/G/P-PLE-0 % and B/G/P-PLE-7 %, respectively. For gelatin only, polylactic acid and polybutylene adipate-co-terephthalate films were prepared by casting gelatine film-forming solution (GFFS), polylactic acid film-forming solution (PFFS) and polybutylene adipate-co-terephthalate film-forming solution (BFFS) on a plastic (GFFS) and glass Petri plates (PFFS and BFFS) with solid content of 4 % (*m*/*V*).

### Study on the extension of shelf-life of fish meat powder using three-layer film with and without Physalis leaf extract

#### Preparation of fish meat powder

Fresh fish (mackerel) fillets were obtained from Pulicat landing centre, Tiruvallur, India, and dried (60 °C for 10 h) in a tray dryer (Everflow Scientific Instruments, Chennai, India). The dried sample was then uniformly ground using a blender.

#### Storage of fish meat powder with and without different films

Fish meat powder (25 g) was sealed in cylindrical aluminium cups (diameter 30 mm), which was covered with PE, polylactic acid, polybutylene adipate-co-terephthalate and gelatine films, with polylactic acid and polybutylene adipate-co-terephthalate on the exposed side of three-layer film without (P/G/B-PLE-0 % and B/G/P-PLE-0 %) and with incorporated (P/G/B-PLE-7 % and B/G/P-PLE-7 %) 7 % of *Physalis* leaf extract. The rubber gaskets and silicone vacuum grease were used to seal the cups. Samples were stored at 27-30 °C ((25±5) % RH). For the control, a sample was kept in uncovered aluminium cups. The thickness of PE film was 0.032 mm, while that of the three-layer films was in the range from 0.0485 to 0.0645 mm. The samples were analysed for 30 days with the interval of 5 days. For the evaluation of volatile compounds and colour, the samples stored for 0 and 30 days were used.

### Analyses

#### Moisture content and pH

The moisture content of preserved sample was analysed according to the AOAC method (*25*). The pH of the sample was evaluated as previously described (*26*).

#### Peroxide value

To determine a peroxide value (PV), samples (10 g) were homogenised with chloroform (50 mL) and then filtered. Glacial acetic acid (15 mL) and potassium iodide solution (*φ*=10 %, 10 mL) were added to the filtrate and kept in the dark (10 min). Then, distilled water (50 mL) and starch solution (1 mL) were added (*25*). The released iodine was titrated against Na_2_S_2_O_3_ (0.02 M) until the blue colour disappeared. Chloroform extract (15 mL) was taken in a pre-weighed beaker and the solvent was evaporated in the water bath. The sample was incubated (100 °C) and further cooled in a desiccator. The fat mass deposited in a beaker was determined and the amount of iodine released per gram of fat was analysed and expressed as mmol of peroxide per kg of fat.

#### Thiobarbituric acid reactive substances

Cold trichloroacetic acid, TCA (20 %, 50 mL), was added to 20 g of sample, which was then rinsed with distilled water and filtered. Then, the filtrate was collected. The obtained extract (5 mL) was mixed with 0.01 M thiobarbituric acid (TBA, 5 mL). TCA (10 %, 5 mL) was used as a blank solution. TBA and blank solutions were heated (100 °C for 30 min) (*27*). The absorbance of the sample was measured at 532 nm using a spectrophotometer (model UV-1900; Shimadzu, Kyoto, Japan) and the thiobarbituric acid reactive substances (TBARS) value was expressed in mg malondialdehyde (MDA) per kg of sample.

#### Total volatile base nitrogen

A sample (10 g) was homogenised with perchloric acid (50 mL) to precipitate the protein and followed by centrifugation (centrifuge model R-24; Remi) at 1000×*g* for 5 min to obtain a supernatant. The supernatant (5 mL) was distilled in a Kjeldhal apparatus for 10 min (*25*). The distillate was collected in a digestion tube to which boric acid (10 mL) and an indicator (2-3 drops) were added. The solution was titrated against HCl until the pink colour appeared and the values were expressed as mg/100 g.

#### Colour

Colour of samples was evaluated (*28*) using Chromameter (MiniScan EZ 4000; Hunter Lab, Reston, VA, USA). ΔE* (total difference in colour) was calculated using the following equation:


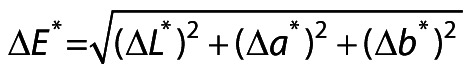
 /1/

#### Sensory analysis

Colour, odour and overall likeness were evaluated by 20 untrained panellists (10 males and 10 females within the age group of 21-32) who were familiar with the fish (mackerel) consumption. They used a 9-point hedonic scale (*29*), with 1=dislike extremely, 5=neither like nor dislike, 9=like extremely. The test was conducted on days 0, 15 and 30 of storage.

#### Analysis of volatile compounds

The volatile compounds in the samples on day 0 and the samples that were covered with PE, P/G/B-PLE-7 % and control (uncovered) film stored for 30 days were measured using a solid-phase microextraction gas chromatography mass spectrometry (SPME-GCMS) (*30*).

### Statistical analyses

A one-way ANOVA was performed and the mean values were analysed using Duncan’s multiple range test and SPSS software v. 26.0 (*31*).

## RESULTS AND DISCUSSION

### Changes in moisture content of fish meat powder covered with different films during storage

**Fig. 1:**
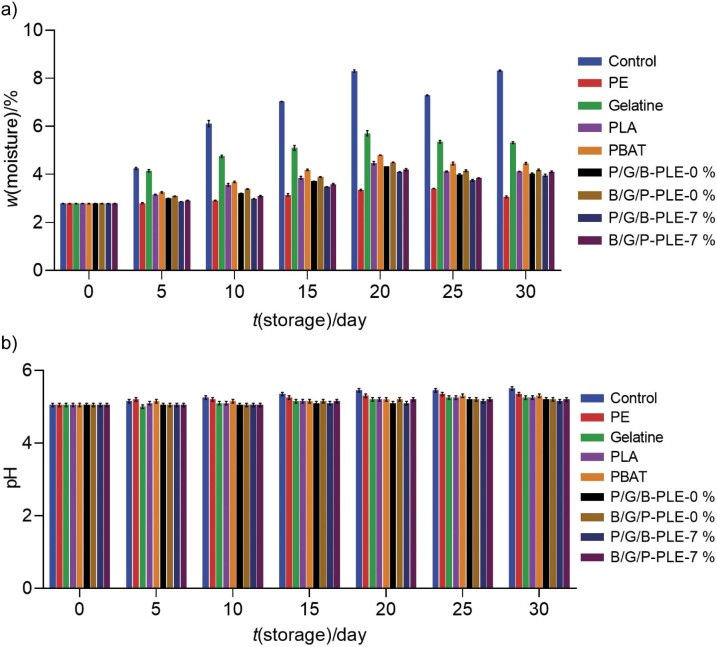
Results for: a) moisture content and b) pH of fish meat powder covered with different films during storage at 27–30 °C for 30 days. Control=uncovered sample, PE=polyethylene, Gelatine=gelatine film, PLA=polylactic acid film, PBAT=polybutylene adipate-co-terephthalate film, P/G/B-PLE-0 % and B/G/P-PLE-0 %=three-layer film without the addition of 7 % *Physalis* leaf extract (PLE) with different layers (PLA and PBAT) on the exposed side, P/G/B-PLE-7 % and B/G/P-PLE-7 %=three-layer film with added 7 % PLE with different layers (PLA and PBAT) on the exposed side

The moisture content of fish meat powder was preserved with different films during 30 days of storage (27–30 °C) (Fig. 1a). The moisture mass fraction of the samples on day 0 was 2.78 %. Samples preserved with films had lower water absorption rate than the control sample throughout the storage, irrespective of the film type (p<0.05). However, the higher moisture mass fraction was found in sample preserved with gelatine film (p<0.05). In three-layer films, the presence of polylactic acid and polybutylene adipate-co-terephthalate could prevent the water molecules from penetrating into the film matrix. This was probably due to the hydrophobicity of the added bioplastics (*32*). In contrast, gelatine film had poor resistance against water due to its hydrophilicity (*33*). Cervera *et al.* (*34*) stated that the higher moisture content found in hydrophilic film was probably due to the interactions between the water vapour and hydrophilic polymer matrix. Therefore, the permeability of water vapour through the matrix of gelatine film increased. Generally, moisture mass fraction in all samples increased due to the higher moisture absorption from the environment through the packing material (*34*). The samples covered with the polylactic acid side (P/G/B-PLE-0 % and P/G/B-PLE-7 %) of three-layer films had lower moisture mass fraction than those covered with the polybutylene adipate-co-terephthalate side (B/G/P-PLE-0 % and B/G/P-PLE-7 %), irrespective of the addition of the leaf extract. In general, the sample covered with P/G/B-PLE-7 % had the lowest moisture mass fraction during the 30 days of storage compared to other bio-based films. This was probably due to the well-organized film matrix formed by the effective H-bonding between the -OH group of the leaf extract and the polymeric chains, creating a ‘tortuous pathway’ (*35*). However, the lowest moisture mass fraction was observed in the sample covered with PE during 30 days of storage. PE had better resistance to water than the protein-based films (*36*). Thus, the findings indicated that the water vapour permeability (WVP) of fish gelatine film was improved by the presence of bioplastics and leaf extract.

### Changes in the pH of fish meat powder covered with different films during storage

The pH of fish meat powder was preserved with different films during 30 days of storage (27–30 °C) (Fig. 1b). The pH of all samples gradually increased during storage. However, the highest pH was recorded for the control sample during the entire storage period (p<0.05), while the lowest was found for the samples preserved with gelatine-based films. Similar results of lower pH were reported for fish slices packed with gelatine-based film (*22*). The formation of alkaline substances (ammonia) and the enzymatic activities of microbes were responsible for higher pH (*37*). Microbiological quality of meat/meat powder was directly correlated with the pH (*38*). A high pH value indicated that the meat powder was probably contaminated with spoilage organisms. Therefore, the samples covered with the enhanced three-layer films had better quality than the control and samples covered with gelatine film.

### Changes in the peroxide value of fish meat powder covered with different films during storage

**Fig. 2:**
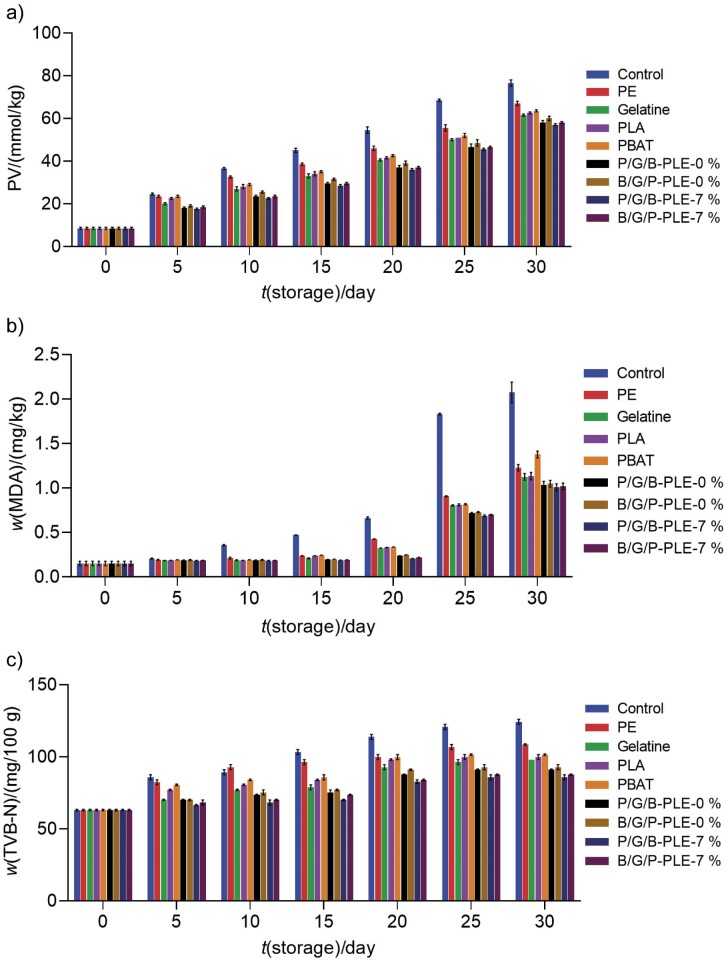
The results for: a) peroxide value (PV), b) thiobarbituric acid reactive substances (TBARS) and c) total volatile basic nitrogen (TVB-N) of fish meat powder covered with different films during storage at 27–30 °C for 30 days. Control=uncovered sample, PE=polyethylene, Gelatine=gelatine film, PLA=polylactic acid film, PBAT=polybutylene adipate-co-terephthalate film, P/G/B-PLE-0 % and B/G/P-PLE-0 %=three-layer film without the addition of 7 % *Physalis* leaf extract (PLE) with different layers (PLA and PBAT) on the exposed side, P/G/B-PLE-7 % and B/G/P-PLE-7 %=three-layer film with added 7 % PLE with different layers (PLA and PBAT) on the exposed side

The lipid oxidation is an important chemical indicator to assess the quality of fish. The peroxide value (PV) of samples preserved with different films during 30 days of storage (27–30 °C) was tested. PV of all samples was found to be 8.5 mmol/kg on day 0 of storage (Fig. 2a). Most fish smell rancid when PV is above 20 mmol/kg. The PV values of fresh fish should be below 10 mmol/kg (*39*). In general, PV of all products gradually increased during storage and it confirmed the synthesis of primary lipid oxidation products (*40*). The free radical formation can induce the formation of hydroperoxides in the oxygen-rich environment. Hydroperoxides are the primary oxidation products that initiate the oxidative changes in mackerel meat powder (*41*). The sample covered with P/G/B-PLE-7 % film yielded the lowest PV amongst all films during the storage period of 30 days. This was mostly due to the complex formation between the phenolic compounds of the leaf extract and gelatine in the film structure. However, the control sample had the highest PV throughout the storage. Gelatine had an excellent oxygen barrier property that was better than that of the commercial PE film (*19*). Thus, the gelatine-based films led to lower PV of fish meat powder. Previous reports found lower PV of the samples preserved with gelatine and gelatine-based films (*36*, *42*). Abreu *et al.* (*43*) found that the blue shark (*Prionace glauca*) samples coated with films containing barley husks had lower PV than their control counterpart. Similar observations of reduced PV were reported for polylactic acid films with added green leaf extract (*20*). Notably, the polylactic acid as an outer layer prevented the water vapour permeability better than polybutylene adipate-co-terephthalate side (*7*) and thus samples covered with P/G/B-PLE-0 and 7 % had lower PV than the samples covered with B/G/P/PLE-0 and B/G/P-PLE-7 %. Thus, the addition of *Physalis* leaf extract to gelatine-based films effectively prevented the primary lipid oxidation and consequently reduced rancidity in fish products.

### Changes in TBARS value of fish meat powder covered with different films during storage

TBARS values of samples preserved with different films during 30 days of storage (27-30 °C) are shown in Fig. 2b. Malondialdehyde (MDA) is a primary aldehyde produced during lipid oxidation of food and expressed as TBARS. It is involved in the breakdown of PUFA in hydroperoxides produced during the oxidation of lipids (*44*). The highest TBARS value was found in control sample, irrespective of storage days (p<0.05). TBARS value is commonly used to evaluate the secondary lipid oxidation in meat and meat products (*45*). The major products of secondary lipid oxidation had been identified as alcohols and aldehydes (*46*). Sample covered with P/G/B-PLE-7 % had the lowest TBARS value compared to other gelatine-based films and it was lower than that of gelatine film (*w*(MDA)=0.183 and 1.007 mg/kg on days 5 and 30, respectively) (p<0.05). However, the TBARS values of samples preserved in PE and bioplastics (polylactic acid and polybutylene adipate-co-terephthalate) were higher. Siripatrawan and Noipha (*47*) found that the pork sausages packed with the chitosan film incorporated with green tea extract had lower TBARS value than the control sample. The higher TBARS value was probably found due to the dehydration of the sample and the enhanced lipid oxidation (*48*). Lower TBARS values can be associated with the depletion of volatile lipid oxidation products, particularly those with lower molecular mass. Lipid oxidation can be accelerated by various mechanisms, including singlet oxygen production, the presence of reactive oxygen species and the enzymatic or non-enzymatic generation of free radicals (*49*). The potential of gelatine film was crucial to serve as a gas barrier that prolonged the shelf life of food (*50*). In contrast, polymeric films with hydrophobic nature (polylactic acid, polybutylene adipate-co-terephthalate and PE) had low oxygen barrier ability, but excellent water vapour barrier. Nevertheless, protein-based films were impermeable to oxygen (*3*). Zanardi *et al.* (*51*) found that the acceptable limit of TBA, expressed as *w*(MDA), was less than 2 mg/kg. On day 30 of storage, the TBARS value of the samples covered with different films was within permissible limits (p<0.05), except for the control sample. Incorporation of polyphenol-rich *Physalis* leaf extract probably induced protein cross-linking in the gelatine film and led to a more compact structure. This may limit the oxygen migration through the film more effectively, as evidenced by the lower rate of lipid oxidation. Therefore, the obtained results suggest that the three-layer gelatine-based film incorporated with *Physalis* leaf extract reduced the secondary lipid oxidation and preserved the quality of product at an acceptable level.

### Changes in TVB-N content of fish meat powder covered with different films during storage

TVB-N content of samples preserved with different films during 30 days of storage (27–30 °C) is shown in Fig. 2c. Generally, fish products easily break down during storage into TVB-N or volatile bases with unpleasant odour that include nitrogen compounds such as ammonia, amines and other volatile bases (*52*). The enzymatic activity and microbiological growth are clearly related to the accumulation of TVB-N (*53*). TVB-N mass fraction of all samples on day 0 was 63 mg/100 g, indicating the freshness of fish meat powder. TVB-N mass fraction of 50 to 70 mg/100 g is reported as a maximum acceptable/edible limit and the fish with the value exceeding this limit is considered as inedible. However, for salted and dried fish, the value should not be greater than 100-200 mg/100 g (*39*). TVB-N content of all samples increased with the storage time (p<0.05). The rate of increase in TVB-N content varied among samples covered with different films. A rapid increase was observed in the control sample throughout the storage, with a maximum value of 124.25 mg/100 g among all samples on day 30. This value was comparably higher than in the samples preserved with gelatine-based films during 30 days of storage (p<0.05). The sample preserved with P/G/B-PLE-7 % had the lowest TVB-N mass fraction (85.75 mg/100 g). This lower TVB-N mass fraction was likely due to more rapid decrease in bacterial population/lower ability of bacteria to oxidatively de-aminate non-protein nitrogen compounds. Similar results were obtained for the samples preserved with gelatine-based films (*21*, *22*). Thus, the findings suggest that three-layer film with incorporated *Physalis* leaf extract slowed down the formation of volatile basic nitrogen compounds and inhibited microbial growth.

### Changes in colour of fish meat powder covered with different films during storage

L*, a*, b* and ΔE* values of samples preserved with various films on day 30 (Table 1) increased compared to those found on day 0 of storage. Lightness of control sample increased on day 30 of storage compared to that on day 0. The lowest a* value (redness) was obtained on day 30 for the sample preserved with PE (p<0.05). The control (uncovered) sample, those preserved with PE and gelatine films had higher yellowness (b* value) than other samples (p<0.05). The increased b* value was likely due to the Maillard reaction and increased lipid oxidation, especially for the control sample (*50*). Nagarajan *et al.* (*19*) reported that glycation of carbonyl groups such as aldehydes produced during lipid oxidation with amino groups of proteins can cause non-enzymatic browning. The increased PV and TBARS values of samples suggest that the browning is probably a result of the increased lipid oxidation. Similar increase of a* and b* values was found in the control sample (*19*, *50*). Salmon meat preserved with gelatine-lignin film had better L*, a* and b* values throughout the storage than the control sample (*54*). The total colour difference (ΔE* value) of samples increased on day 30. This result is in agreement with the lower lightness (L* value) of samples preserved with bioplastics and three-layer films. Therefore, the films had an impact on the colour of the samples that was most likely related to the lipid oxidation and Maillard reaction.

**Table 1:** Colour of fish meat powder covered with different films during storage at 27–30 °C for 0 and 30 days

Sample	L*	a*	b*	ΔE*
*t*(storage)=0 day				
	50.3±0.4	9.7±0.2	34.5±0.2	56.2±0.5
*t*(storage)=30 day				
Control	(53.0±0.5)^a^	(11.71±0.07)^a^	(39.4±0.9)^a^	(57.8±1.2)^a^
PE	(49.5±0.6)^cde^	(10.0±0.7)^c^	(39.2±5.7)^a^	(60.0±4.1)^a^
Gelatine	(51.5±0.4)^b^	(11.6±0.2)^a^	(38.62±0.08)^ab^	(58.33±0.4)^a^
PLA	(48.6±0.8)^e^	(10.99±0.09)^b^	(35.2±0.3)^b^	(58.2±0.4)^a^
PBAT	(49.7±0.6)^cd^	(10.92±0.07)^b^	(35.50±0.02)^ab^	(57.6±0.4)^a^
P/G/B-PLE-0 %	(48.9±0.8)^de^	(10.9±0.1)^b^	(37.5±1.9)^ab^	(59.4±1.7)^a^
B/G/P-PLE-0 %	(49.8±0.3)^cd^	(11.37±0.06)^ab^	(36.85±0.04)^ab^	(58.4±0.2)^a^
P/G/B-PLE-7 %	(49.6±0.8)^cde^	(11.32±0.02)^ab^	(36.2±0.1)^ab^	(58.1±0.6)^a^
B/G/P-PLE-7 %	(50.55±0.02)^bc^	(11.46±0.04)^a^	(36.6±0.3)^ab^	(57.7±0.2)^a^

The values are mean±SD (*N*=3). Different lower-case letters in superscript in the same column indicate significant differences on day 30 of storage (p<0.05). Control=uncovered sample, PE=polyethylene, Gelatine=gelatine film, PLA=polylactic acid film, PBAT=polybutylene adipate-co-terephthalate film, P/G/B-PLE-0 % and B/G/P-PLE-0 %=three-layer film without the addition of 7 % *Physalis* leaf extract (PLE) with different layers (PLA and PBAT) on the exposed side, P/G/B-PLE-7 % and B/G/P-PLE-7 %=three-layer film with added 7 % PLE with different layers (PLA and PBAT) on the exposed side

### Changes in sensory properties of fish meat powder covered with different films during storage

**Fig. 3:**
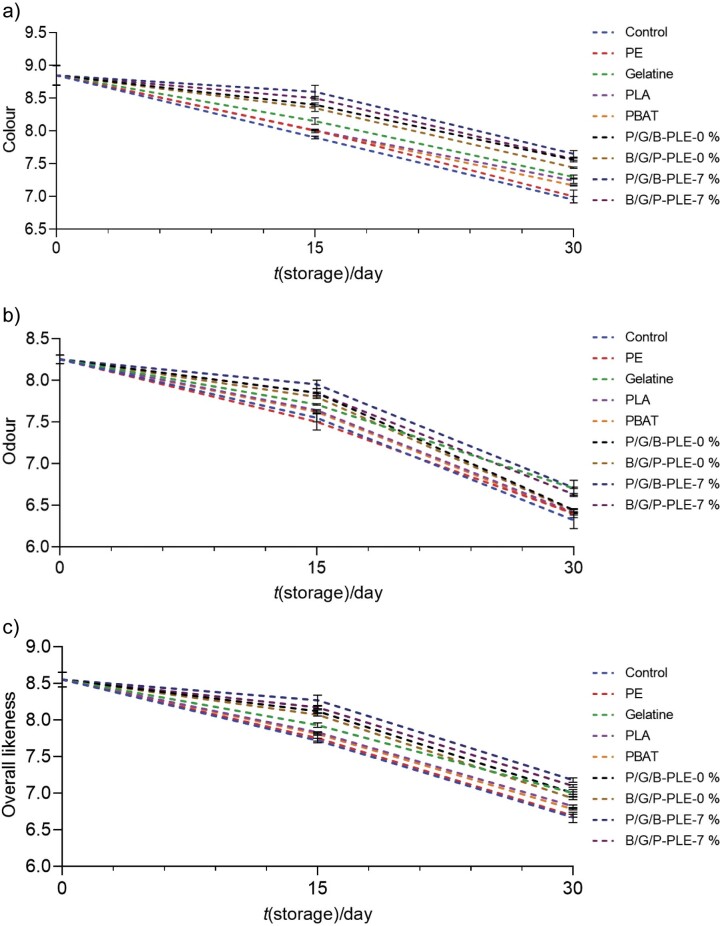
Sensory analysis: a) colour, b) odour and c) overall likeness of fish meat powder covered with different films during storage at 27–30 °C for 0, 15 and 30 days. Control=uncovered sample, PE=polyethylene, Gelatine=gelatine film, PLA=polylactic acid film, PBAT=polybutylene adipate-co-terephthalate film, P/G/B-PLE-0 % and B/G/P-PLE-0 %=three-layer film without the addition of 7 % *Physalis* leaf extract (PLE) with different layers (PLA and PBAT) on the exposed side, P/G/B-PLE-7 % and B/G/P-PLE-7 %=three-layer film with added 7 % PLE with different layers (PLA and PBAT) on the exposed side

The scores for colour, odour and overall likeness were lower for control sample (Fig. 3) during storage for 30 days (p<0.05). The deteriorating sensory properties of control (uncovered) sample indicated by the production of off-odour with colour changes after 15 days of storage were likely due to the microbial growth and lipid oxidation. The sample covered with P/G/B-PLE-7 % had higher scores than other samples. Gelatine and three-layer films with and without *Physalis* leaf extract had better overall likeness than other films (p<0.05). This can be due to the barrier ability of gelatine films to oxygen. This is confirmed by the lower PV and TBARS values (Fig. 2a and Fig. 2b, respectively) for the samples covered with gelatine-based films, especially P/G/B-PLE-7 %. The addition of *Physalis* leaf extract improved the odour, colour and overall quality of the fish meat powder over the storage period of 30 days. Notably, incorporation of oregano oil (0.05 %, *V*/*m*) resulted in better likeness score and it increased the shelf-life of fish from 11 to 26 days (*55*). Thus, the three-layer film incorporated with *Physalis* leaf extract could minimize lipid oxidation and thus prolong the shelf-life of fish products.

### Changes in volatile compounds of fish meat powder covered with the selected films during storage

The volatile compounds of samples on day 0 and the samples preserved with the three-layer (P/G/B-PLE-7 %) film and the PE film compared to the control (uncovered) sample after storage for 30 days are shown in Table 2. Fish meat powder contained volatile compounds including aldehydes (benzaldehyde, nonanal, octanal and hexanal) and alcohols (1-pentanol, ethanol, 1-octene-3-ol, 1-octanol, 1-heptanol, 2-penten-1-ol and 1-penten-3-ol) on day 0. Among alcohols, the abundance of 1-penten-3-ol and 1-heptanol was found to be 17.48 and 15.18·10^7^, respectively, and among aldehydes, benzaldehyde (42.81·10^7^) and nonanal (16.08·10^7^) were found on day 0. Aldehydes have low threshold values, are the primary contributors to off-flavour and serve as the key indicators of lipid oxidation (*36*). The numerous aldehydes such as nonanal, octanal, pentanal and hexanal develop during oxidation (*36*). Hexanal and benzaldehyde were the two most prevalent aldehydes in the samples, followed by nonanal and octanal. Nagarajan *et al.* (*19*) reported that both hexanal and nonanal were secondary oxidation products of linolenic acid. Hexanal is a good indicator of rancidity in fish meat, as it was detected in the control samples and those covered with PE film on day 30. However, the lowest amount was observed in the sample covered with P/G/B-PLE-7 %. Similar results were reported for fish meat powder preserved with gelatine-based nanocomposite film containing ethanolic extract from coconut husk (*19*). The presence of aldehydes such as nonanal and octanal was not detectable in the meat powder covered with P/G/B-PLE-7 % on day 30. Aldehydes were most abundant in the control sample. Haemoglobin-catalysed nonanal was the cause of the oxidized oil odour. The presence of carbonyl compounds, *e.g.* octanal, is responsible for fishy odour (*19*). However, the volatile compounds such as nonanal and octanal disappeared on day 30. Alcohols are the secondary by-products of the breakdown of hydroperoxide. Ethanol was not detectable on day 0 but it was found on day 30. Lower amount of ethanol was found in the sample covered with P/G/B-PLE-7 % film than in other samples. Benzaldehyde was detected in all samples on day 30 of storage, although the sample covered with P/G/B-PLE-7 % film had lower amount. The maximum amount of 1-penten-3-ol, 1-octene-3-ol and 2-penten-1-ol was found in the control sample, while after 30 days of storage, the sample preserved with P/G/B-PLE-7 % film had the lowest amount. The compounds such as 1-octanol, 1-heptanol and 1-pentanol were not detected on day 30. This was probably due to their volatilisation during storage. Aliphatic alcohols like 1-octene-3-ol and 1-pentene-3-ol, responsible for musty flavour, arise from lipid oxidation (*19*). All fish species contain C8-alcohols and fatty acid hydroperoxides decompose to form 1-alkanals (*e.g*. hexanal and pentanol) and 1-alkanols (*19*).

**Table 2:** Volatile compounds of fish meat powder covered with selected films during storage at 27–30 °C for 0 and 30 days

	*t*(storage)/day			
Volatile compound	0	30		
		Control	PE	P/G/B-PLE-7 %
Hexanal	ND	28.43	27.30	1.83
Nonanal	16.08	ND	ND	ND
Octanal	10.82	ND	ND	ND
Benzaldehyde	42.81	37.43	26.11	15.36
Ethanol	ND	55.87	43.29	28.02
1-Heptanol	15.18	ND	ND	ND
1-Octanol	6.42	ND	ND	ND
1-Octene-3-ol	ND	30.84	27.48	4.13
1-Pentanol	9.87	ND	ND	ND
1-Penten-3-ol	17.48	79.47	64.28	58.44
2-Penten-1-ol	7.41	21.78	20.18	14.49
2,3-Dimethyl-5-ethyl pyrazine	ND	60.19	52.42	21.62
2,3,5-Trimethyl pyrazine	ND	161.42	152.18	90.63
2,3,5,6-Tetramethyl pyrazine	ND	532.42	518.34	201.84
Butanoic acid	ND	541.67	218.40	108.69
3-Methyl-butanoic acid	ND	651.42	235.89	52.69
Propanoic acid	ND	64.23	18.39	12.45
Heptane	ND	41.08	41.02	40.14

ND=not detectable. Control=uncovered sample, PE=polyethylene, P/G/B-PLE-7 %=three-layer film with added 7 % *Physalis* leaf extract and polylactic acid on the exposed side

Different amounts of acids (butanoic, propanoic and 3-methyl-butanoic acid) and alkyl pyrazine (2,3,5-trimethyl, 2,3,5,6-tetramethyl and 2,3-dimethyl-5-ethyl pyrazine) were also detected in the samples, depending on the film used. Alkyl pyrazines are pyrazine-based compounds with various patterns of substitution. The naturally occurring, strong aromatic alkyl pyrazines typically have an extremely low threshold for odour. In general, alkyl pyrazines are produced during cooking of a variety of foods *via* Maillard reactions (*36*). The pyrazines were detected in higher amounts in the control sample and those covered with the PE film and P/G/B-PLE-7 % film after 30 days of storage. Heptane was not detectable on day 0 of storage, but it was detected on day 30 with the highest value of 41.08·10^7^ in the control and the lowest (40.14·10^7^) in the sample covered with P/G/B-PLE-7 % film. Samples covered with any film had lower amounts of all acids. In general, TBARS values correlate well with the results for volatile compounds. Therefore, it was confirmed that the films made from fish skin gelatine with added bioplastics and 7 % *Physalis* leaf extract could delay oxidative degradation and inhibit the development of unpleasant odour in fish meat powder by blocking the passage of gas, light and water vapour.

## CONCLUSION

Three-layer film incorporated with 7 % *Physalis* leaf extract can effectively reduce the moisture permeation and lipid oxidation of fish meat powder compared to the samples preserved with polyethylene (PE), polylactic acid (P), gelatine (G) and polybutylene adipate-co-terephthalate (B) films and control (uncovered) sample. The three-layer (P/G/B) film incorporated with 7 % leaf extract significantly reduced the deterioration by maintaining the quality of fish meat powder, as confirmed by lower peroxide value, TBARS and TVB-N values. Polylactic acid, gelatine and polybutylene adipate-co-terephthalate layers of the developed three-layer films can serve as the external (outer layer), middle and internal (in contact with food) layers, respectively, for food packaging. Thus, the three-layer film with optimal amount (7 %) of leaf extract can gradually replace the non-biodegradable plastic films in near future.

## Data Availability

Data were not shared.
